# wgd—simple command line tools for the analysis of ancient whole-genome duplications

**DOI:** 10.1093/bioinformatics/bty915

**Published:** 2018-11-06

**Authors:** Arthur Zwaenepoel, Yves Van de Peer

**Affiliations:** 1 Department of Plant Biotechnology and Bioinformatics, Ghent University; 2 Center for Plant Systems Biology, VIB; 3 Bioinformatics Institute Ghent, Ghent University, Ghent, Belgium; 4 Department of Biochemistry, Genetics and Microbiology, University of Pretoria, Pretoria, South Africa

## Abstract

**Summary:**

Ancient whole-genome duplications (WGDs) have been uncovered in almost all major lineages of life on Earth and the search for traces or remnants of such events has become standard practice in most genome analyses. This is especially true for plants, where ancient WGDs are abundant. Common approaches to find evidence for ancient WGDs include the construction of *K*_S_ distributions and the analysis of intragenomic colinearity. Despite the increased interest in WGDs and the acknowledgment of their evolutionary importance, user-friendly and comprehensive tools for their analysis are lacking. Here, we present an easy to use command-line tool for *K*_S_ distribution construction named wgd. The wgd suite provides commonly used *K*_S_ and colinearity analysis workflows together with tools for modeling and visualization, rendering these analyses accessible to genomics researchers in a convenient manner.

**Availability and implementation:**

wgd is free and open source software implemented in Python and is available at https://github.com/arzwa/wgd.

**Supplementary information:**

Supplementary data are available at *Bioinformatics* online.

## 1 Introduction

In this era of whole-genome sequencing, many ancient whole-genome duplication (WGD) events have been uncovered across the eukaryotic tree of life (Van de Peer *et al.*, 2017). One of the main approaches for revealing ancient WGDs using genomic data is the construction of whole paranome *K*_S_ distributions (e.g. Blanc and Wolfe, 2004; Cui *et al.*, 2006; Lynch and Conery, 2000; Vanneste *et al.*, 2013), where *K*_S_ is the synonymous distance or the estimated number of synonymous substitutions per synonymous site. Under the assumption of neutral evolution at synonymous sites, the synonymous distance between two coding sequences serves as a proxy for the divergence time of two sequences. Under a model of continuous small-scale gene duplication (SSD) and loss of duplicated copies not under selection, a whole paranome *K*_S_ distribution is expected to show an exponential decay of the number of retained duplicates in function of age (Blanc and Wolfe, 2004; Lynch and Conery, 2000). Against this background of SSDs, large-scale duplication events, such as WGDs, are visible as peaks in the number of retained duplicates at a particular age.

Several issues compromise the use of *K*_S_ distributions for WGD inference, and these were extensively addressed in Vanneste *et al*. (2013). When high-quality genome assemblies are available, gene colinearity (often called synteny) based analyses may further aid in unveiling WGDs or large segmental duplications (Van de Peer, 2004). WGDs are expected to leave large blocks with high intragenomic colinearity, and paralogs located in such colinear segments (anchor pairs) can therefore be traced back more reliably to a particular event, enabling their use for downstream analyses such as molecular dating (Vanneste *et al.*, 2014) or functional analysis.

While these methods have been used frequently in genomics research, no comprehensive and user-friendly software is available to perform these analyses, and researchers have often resorted to custom pipelines. Here, we fill this gap with an integrated suite for *K*_S_ and colinearity based analysis of ancient WGDs. We briefly discuss the methods implemented here, but refer to the documentation and Supplementary Material for more information.

## 2 Materials and methods

### 2.1 Gene family delineation

Delineation of paralogous gene families and one-to-one orthologs starts from all-versus-all BLASTp similarity searches or precomputed BLAST results and is performed using ‘wgd mcl’. For whole paranome delineation, MCL (van Dongen, 2000) is then used to cluster sequences in paralogous gene families. One-to-one orthologs are determined using the commonly employed reciprocal best hit strategy.

### 2.2 *K*_S_ distribution construction

A *K*_S_ distribution for a set of paralogous families or one-to-one orthologs can be constructed using the ‘wgd ksd’ subcommand, and we closely follow the approach used by Vanneste *et al*. (2013). We refrain from a full description of the methodology here and refer to the Supplementary Material instead.

### 2.3 Colinearity analyses

**Fig. 1.**
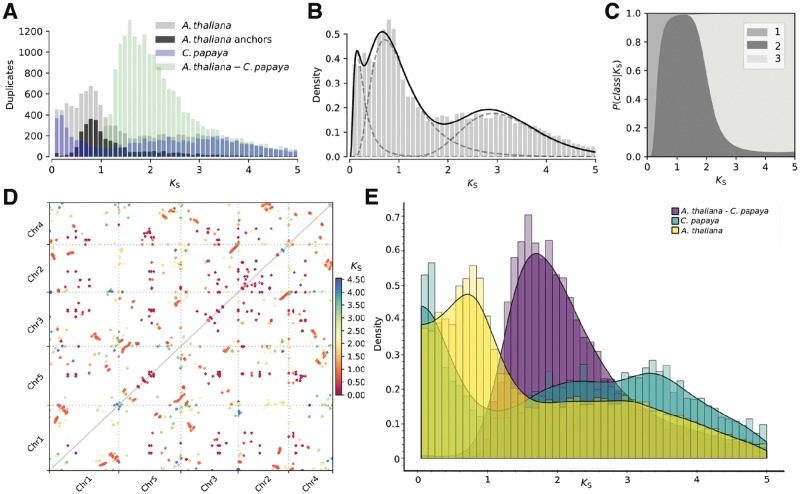
Illustration of the various tools and visualizations in wgd. (**A**) *Arabidopsis thaliana* and *Carica papaya* paranome *K*_S_ distributions overlayed with the *K*_S_ distribution of anchor pairs for *A. thaliana* and *K*_S_ distribution of one-to-one orthologs of *C. papaya* and *A. thaliana.* (**B**) Mixture of three log-normal distributions fitted to the *K*_S_ distribution of *A. thaliana*, using the Variational Bayes algorithm with *γ = *10^−3^. (**C**) Plot showing the probability to belong to a particular component of the mixture shown in (B) in function of *K*_S._ These probabilities can be used to define component-wise paralogs for further downstream analyses. (**D**) *K*_S_-colored dotplot for *A. thaliana*, showing colinear blocks identified by I-ADHoRe, colored by their median *K*_S_ value. (**E**) Interactive histogram visualization (user interface not shown, see Supplementary Fig. S1), showing the whole paranome *K*_S_ distributions using histograms and kernel density estimates for *A. thaliana* and *C. papaya* together with the *K*_S_ distribution of one-to-one orthologs in these species. We refer to the Supplementary Material for detailed methods

When high-quality structural genome annotations are available, the ‘wgd syn’ tool allows the identification of intragenomic colinear blocks and their corresponding anchor pairs using I-ADHoRe 3.0 (Proost *et al.*, 2012). Whole-genome syntenic dotplots are generated, and if a *K*_S_ distribution is provided, *K*_S_-colored dotplots and anchor pair *K*_S_ distributions are generated (Fig. 1).

### 2.4 Kernel density estimation and mixture modeling

Downstream analyses of *K*_S_ distributions have often consisted in fitting statistical models and visualizing these. We provide tools (‘wgd kde’) for fitting kernel density estimates (KDEs). Importantly, we apply a correction for boundary effects, which are often neglected but may lead to artificial peaks in low *K*_S_ regions. As peaks derived from WGDs are expected to be approximately log-normally distributed, Gaussian mixture models (GMMs) have also been used frequently to analyze *K*_S_ distributions. We provide tools (‘wgd mix’) for fitting mixtures of log-normal components using different inference algorithms, implemented using the scikit-learn python library (Pedregosa *et al.*, 2011). Common approaches to determine the optimal number of components are provided, using the Akaike or Bayesian information criterion, however we would like to warn prospective users to carefully interpret ‘significant’ components, as these GMMs may strongly overfit the empirical distribution (Tiley *et al.*, 2018).

### 2.5 Interactive visualization

Lastly, we provide tools for (interactive) visualization of histograms and KDEs in ‘wgd viz’ (Fig. 1). These tools allow visualization of multiple *K*_S_ distributions for comparative purposes as well as modification of key visualization parameters such as the histogram bin-width or the KDE bandwidth. We encourage researchers to modify and explore the influence of these to guide careful analysis of the distributions and to prevent misinterpretations of KDE or histogram artifacts as biologically interesting features.

## 3 Conclusion

We provide, to our knowledge, the first comprehensive toolshed for *K*_S_ and colinearity based analysis of WGDs in an easy to use and freely available package named wgd. We hope that, besides being a useful tool for researchers, it will also aid in preventing common pitfalls and misinterpretations when analyzing putative WGDs in genomic data.

## Funding

This work was supported by the European Union Seventh Framework Programme (FP7/2007-2013) under European Research Council Advanced Grant Agreement 322739—DOUBLEUP [to Y.V.d.P]; and a PhD Fellowship of the Research Foundation—Flanders (FWO) [to A.Z.].

*Conflict of Interest*: none declared.

## Supplementary Material

bty915_Supplementary_Data
