# Supplementary material for: wgd—simple command line tools for the analysis of ancient whole-genome duplications
Source: Bioinformatics. 2018 Nov 6;35(12):2153–5. doi: 10.1093/bioinformatics/bty915 (PMC6581438; doi:10.1093/bioinformatics/bty915)
Supplement: bty915_Supplementary_Data [file bty915_supplementary_data.pdf]

Supplementary material wgd - simple command line tools for the analysis of ancient whole genome duplications

Arthur Zwaenepoel & Yves Van de Peer

1. Introduction

Here we supply additional methodological details with regard to the wgd software package for the analysis of whole genome duplications (WGDs) in genome data. In section 5, we provide a detailed step-by-step protocol to acquire the results shown in Figure 1 of our main paper. In that section, we also show exactly which parameter settings were used to generate the relevant data.

2. K_S distribution construction

We describe the workflow for paralogous families, as the approach for one-vs.-one ortholog K_S distributions is essentially the same. For every gene family, a multiple sequence alignment (MSA) is inferred using one of the supported aligners (currently MUSCLE (Edgar 2004) and MAFFT (Katoh *et al.* 2013) are supported for protein MSAs (which are back-translated), and PRANK (Löytynoja & Goldman 2008) for codon-level MSAs). Subsequently, the K_S values for all gene pairs in the family are estimated by maximum-likelihood (ML) using the model of Nielsen & Yang (1998) as implemented in the codeml program from the PAML package (Yang 2007). Codon frequencies are determined using the F3X4 method based on the average nucleotide frequencies at the three codon positions. Codon model 0 is used for pairwise K_S, K_A and ω estimation, assuming a constant ω across sites and branches. K_S estimates are subsequently node-weighted to correct for the redundancy in K_S estimates when the family has undergone multiple duplication events. To this end the user can choose to apply average linkage clustering (e.g. Maere *et al.* 2005) or phylogenetic tree construction (e.g. Vanneste *et al.* 2015) using FastTree (Price *et al.* 2010) or PhyML (Guindon *et al.* 2010). We note that the modular design of the package allows to easily provide support for other aligners and phylogenetic tree inference programs than those currently supported. The full K_S analysis workflow can be executed in parallel to allow efficient computation on multi-core systems.

3. Kernel density estimation & Gaussian mixture modeling

Kernel density estimates (KDEs) are used frequently for the visualization of empirical distributions as density curves, and have been used for K_S distributions as well. One problem that is rarely accounted for however when fitting KDEs to K_S distributions is the boundary effect at $K_S = 0$ (or any other lower bound when some filtering step is used). When not accounted for the boundary, a KDE will strongly underestimate the density around the boundary, where the size of this region of underestimation is dependent on the bandwidth. This underestimation may lead to spurious peaks in low K_S regions. One simple approach to account for the boundary effect is to reflect the data around the boundary and generate a new data set from the combination of the original data and the reflected data. One can then fit a KDE to the resulting data set, which is of course visualized in the original K_S range of interest. We note that this approach effectively amounts to the assumption that the derivative of the density curve at the boundary is equal to 0. This was implemented both in `wgd kde` and `wgd viz`. Gaussian mixture modeling has been used frequently in the literature to study K_S distributions (e.g. Barker *et al.* 2008, Vanneste *et al.* 2015, Devos *et al.* 2006, Li *et al.* 2018). However there has been a widespread misconception that peaks in the K_S originating from WGDs are

expected to show a Normal distribution (e.g. Barker *et al.* 2008, Devos *et al.* 2006, Li *et al.* 2018). Simple molecular evolutionary arguments however indicate otherwise. If we consider synonymous substitution as a Poisson process, and synonymous substitution rates for different duplicate pairs sampled from some Gamma distribution, the expected distribution of number of synonymous substitutions will follow a Negative binomial distribution (which is well approximated in the continuous case by the Gamma or log-normal distribution). WGD peaks in the K_S distribution will therefore have positive skew, and this effect will be stronger the more recent the WGD. Normal GMMs are not able to account for this effect, and neither can they cope with the background exponential decay from SSDs. We provide tools for fitting mixtures of log-normal components to K_S distributions using either an expectation-maximization (EM) algorithm or a variational Bayes (VB) inference algorithm (Blei & Jordan 2006). The latter is of particular interest here, as it allows to mitigate to some extent the common overfitting problems encountered with GMMs by means of regularization. The parameter γ governs the strength of regularization, with a small γ leading to stronger regularization, making less components likely to be active in the mixture. Therefore this strategy allows to some degree to automatically select the number of components, as for strong enough regularization the weights of spurious components will be shrunk towards 0. However in practice, overfitting can still be a problem, and active components should be interpreted with caution (see for example Tiley *et al.* (2018) for a recent study on mixture modeling for WGD inference).

4. Comparison of wgd with other available tools

Table 1: Comparison of wgd with some frequently used tools for studying WGDs. We note that of these tools, only wgd is specifically designed for the purpose of providing an integrative tool for WGD analysis, whereas the other tools provide some of the analyses that are often performed when studying WGDs. This list may be inexhaustive but focuses on tools that have been used in recent studies. An 'x' marks a feature as available. Notes: (i) Uses I-AdHoRe 3.0, only for intra-genomic co-linearity analysis in the current version. (ii) Uses the heuristic counting method of Nei & Gojobori (1986), instead of a model of codon substitution. (iii) Does not use clustering approach to identify paralogous families. (iv) Does not perform node-weighting or node-averaging. References; SyMap: Soderlund *et al.* (2011); MCscanX: Wang *et al.* (2012); CoGe: Lyons *et al.* (2008), Haug-Baltzell *et al.* (2017), FastKs: McKain *et al.* (2016). We note that evopipes.net (Barker *et al.* 2010) also provides tools for K_S distribution construction and gene family inference, however this web-based platform has been unavailable for some time at the time of writing.

	wgd	SyMap	CoGe	MCScanX	FastKs
Paranome/one-vs-one ortholog delineation	x				x^{iii}
Whole paranome K_S distributions	x				x^{iv}
One-vs-one ortholog K_S distributions	x				x
Mixture modeling	x				x
Co-linearity dotplots	x^{i}	x	x	x	
Interactive visualizations	x	x	x		
Anchor-pair K_S distributions	x		x	x^{ii}	
Command line interface	x			x	
Web interface		x	x		
Open source	x	x	x	x	x

5. *Arabidopsis thaliana* example recipe

This is a recipe for performing the analyses to obtain the results presented in Figure 1 of the wgd application note. The analyses are presented for a test data set, but the exact same commands can be used for the full data set to acquire the full results. The results (gene families, K_S distributions and anchor pair K_S distributions) can also be found in the example directory of the wgd repository.

First install `wgd`, if you haven't yet, (be sure you have Python3 installed):

```
git clone https://github.com/arzwa/wgd.git
cd wgd
pip install .  # if this doesn't work, try pip3 instead of pip
```

You will also need to have Blast, MUSCLE, FastTree and I-ADHoRe installed for the full analysis.

Get the sequence data from PLAZA (4.0)

```
wget ftp://ftp.psb.ugent.be/pub/plaza/plaza_public_dicots_04//Fasta/cds.ath.fasta.gz
gunzip cds.ath.fasta.gz
```

Note that this annotation contains mitochondrial and chloroplast genes as well as transposable elements. To eliminate these do

```
grep -A 1 ">AT[1-9]G" cds.ath.fasta > cds.ath_filtered.fasta
```

Optional: For a quick test analysis, get a random sample of sequences from the file (skip this if you want to do the full analysis).

```
grep ">" cds.ath_filtered.fasta | shuf | head -n 1000 > ids
grep -A 1 -f ids cds.ath_filtered.fasta | sed "/--/d" > sample.fasta
rm ids
```

Run an all-*vs.*-all Blastp analysis and cluster using MCL, assuming you're working with `sample.fasta` (replace with `cds.ath_filtered.fasta` for a full analysis). We use the default parameters, which are at the moment of writing an e-value cut-off of 10^{-10} and an inflation factor of 2.0.

```
wgd mcl -s sample.fasta --cds --mcl
```

A directory named `wgd_blast` will appear that will contain some gene families in the file `sample.fasta.blast.tsv.mcl`. We move the file to the working directory and give it a shorter name for convenience

```
mv wgd_blast/sample.fasta.blast.tsv.mcl ./sample.mcl
```

Now let's compute a K_S distribution (use `-n` to set the number of cores to use, defaults to 4). We again use default methods, being the family-wise mode (as opposed to the pairwise mode, where codeml is run for every pair instead of the whole family), MUSCLE for multiple sequence alignment and FastTree for node-weighting.

```
wgd ksd sample.mcl sample.fasta
```

This creates a directory `wgd_ksd`. You can check the histograms that were generated or inspect the K_S distribution itself:

```
head -n 3 wgd_ksd/*tsv
```

```
      AlignmentCoverage AlignmentIdentity   ... Ks   Node    Omega   Outlier Paralog1    ...
    AT1G77815__AT3G09510    0.18941 0.50538 ... 2.4193  2.0 0.308   False   AT1G77815   ...
    AT2G27980__AT2G37520    0.73607 0.49256 ... 10.1451 2.0 0.0613  True    AT2G27980   ...
```

To get anchor pairs, first download the GFF file

```
wget ftp://ftp.psb.ugent.be/pub/plaza/plaza_public_dicots_04// # ... omit this line break
        GFF/ath/Arabidopsis_thaliana.COL0.Araport11.longest_transcript.all_features.gff3.gz
gunzip Arabidopsis_thaliana.COL0.Araport11.longest_transcript.all_features.gff3.gz
mv Arabidopsis_thaliana.COL0.Araport11.longest_transcript.all_features.gff3 ath.gff
```

Then run `wgd syn`, again with default parameters (which can be seen in the documentation here: https://wgd.readthedocs.io/en/latest/syn.html#wgd.colinearity.write_config_adhore):

```
wgd syn -ks wgd_ksd/sample.fasta.ks.tsv -f gene -a ID ath.gff sample.mcl
```

Chances are that you get the `WARNING   No multiplicons found!` warning when using the small test data, in that case nothing interesting happens. However when doing the full analysis, you should find dotplots

and anchor-pair K_S distributions in the `wgd_syn` directory. Here you can also find the configuration file for I-ADHoRe (`adhore.conf`) with the exact parameter settings, which in this case was:

```
genome= genome
Chr1 ./wgd_syn/gene_lists/Chr1.lst
Chr2 ./wgd_syn/gene_lists/Chr2.lst
Chr3 ./wgd_syn/gene_lists/Chr3.lst
Chr4 ./wgd_syn/gene_lists/Chr4.lst
Chr5 ./wgd_syn/gene_lists/Chr5.lst
ChrC ./wgd_syn/gene_lists/ChrC.lst
ChrM ./wgd_syn/gene_lists/ChrM.lst
blast_table= ./wgd_syn/families.tsv
output_path= ./wgd_syn/i-adhore-out
gap_size= 30
q_value= 0.75
cluster_gap= 35
prob_cutoff= 0.01
anchor_points= 3
alignment_method= gg2
level_2_only= false
table_type= family
multiple_hypothesis_correction= FDR
visualizeGHM= false
visualizeAlignment= true
```

Mixture models can be fit using the following command:

```
wgd mix --method bgmm wgd_ksd/sample.fasta.ks.tsv
```

which will fit models using the variational Bayes algorithm with up to four components. You can find plots and output data with component-wise probabilities in the directory `wgd mix`.

The *Carica papaya* distribution was obtained with identical methods, whereas a one-*vs.*-one ortholog distribution can be obtained by following the same approach but with the one-*vs.*-one flag in `wgd mcl` and `wgd ksd`. When all K_S distributions are computed, `wgd viz` can be used to interactively visualize these together. To do so, put all distributions in one directory (I assume it is named `ks_dir`) and run a bokeh server instance:

```
bokeh serve &
```

Then run

```
wgd viz -i ./ks_dir
```

A browser window will appear where you can toy around with the visualization. Below (Supplementary figure 1) a screenshot of the current user interface is included, with as example distributions those included in Figure 1 of our main paper.

6. Acknowledgements

We thank Rolf Lohaus for testing the software and providing valuable comments that led towards the improvement thereof.

References

Barker, M.S., Kane, N.C., Matvienko, M., Kozik, A., Michelmore, R.W., Knapp, S.J., and Rieseberg, L.H. (2008). Multiple Paleopolyploidizations during the Evolution of the Compositae Reveal Parallel Patterns of Duplicate Gene Retention after Millions of Years. Mol Biol Evol 25, 2445–2455.

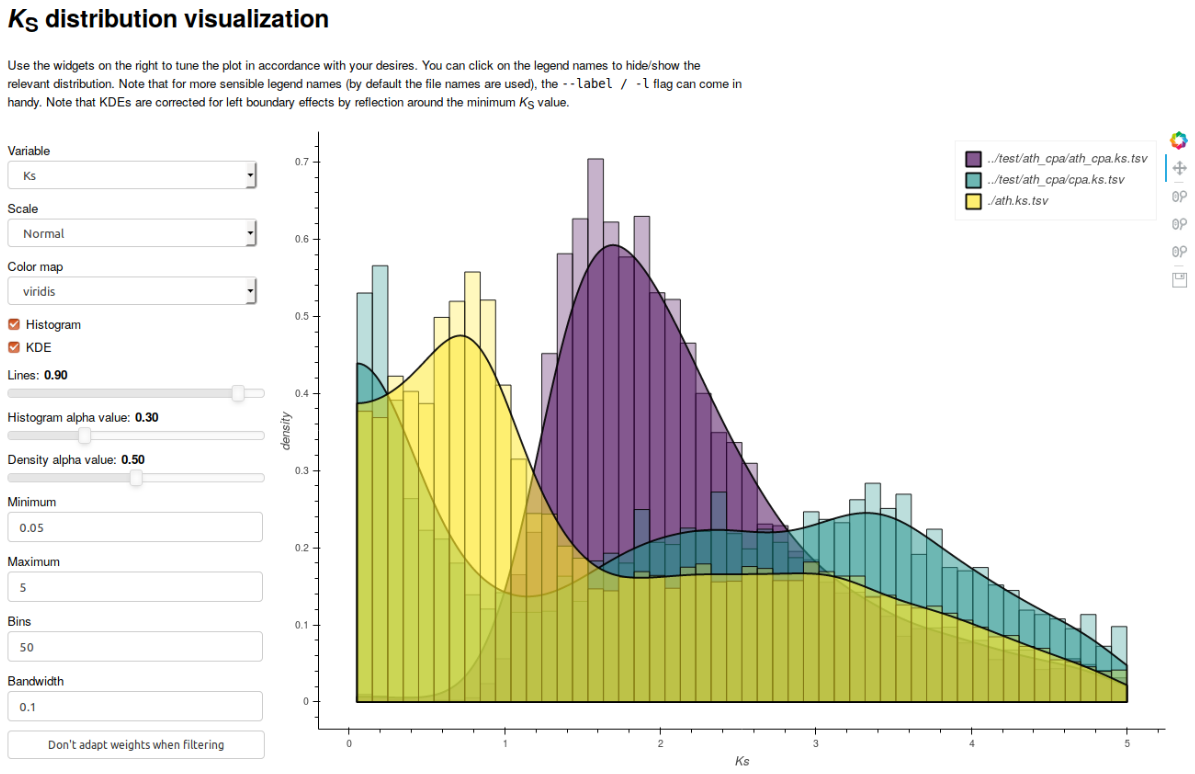

Figure 1: **Screenshot of the current user interface (UI) in `wgd viz` for interactive visualization of K_S distributions and KDEs.** The UI allows modifying the colors, hiding and showing different distributions (by clicking labels in the legend), adapting visual attributes (e.g. opacity of the KDE), filtering and (optional) reweighting by K_S range and modifying the number of histogram bins and KDE bandwidth. The user can scroll and zoom using the mouse and save the plot to a file.

Barker, M.S., Dlugosch, K.M., Dinh, L., Challa, R.S., Kane, N.C., King, M.G., and Rieseberg, L.H. (2010). EvoPipes.net: Bioinformatic Tools for Ecological and Evolutionary Genomics. Evol Bioinform Online 6, 143–149.

Blei, D.M., and Jordan, M.I. (2006). Variational inference for Dirichlet process mixtures. Bayesian Analysis 1, 121–143.

Devos, N., Szövényi, P., Weston, D.J., Rothfels, C.J., Johnson, M.G., and Shaw, A.J. (2016). Analyses of transcriptome sequences reveal multiple ancient large-scale duplication events in the ancestor of Sphagnopsida (Bryophyta). New Phytologist 211, 300–318.

Edgar, R.C. (2004). MUSCLE: multiple sequence alignment with high accuracy and high throughput. Nucleic Acids Res. 32, 1792–1797.

Guindon, S., Dufayard, J.-F., Lefort, V., Anisimova, M., Hordijk, W., and Gascuel, O. (2010). New Algorithms and Methods to Estimate Maximum-Likelihood Phylogenies: Assessing the Performance of PhyML 3.0. Systematic Biology 59, 307–321.

Haug-Baltzell, A., Stephens, S.A., Davey, S., Scheidegger, C.E., and Lyons, E. (2017). SynMap2 and SynMap3D: web-based whole-genome synteny browsers. Bioinformatics 33, 2197–2198.

Katoh, K., and Standley, D.M. (2013). MAFFT Multiple Sequence Alignment Software Version 7: Improvements in Performance and Usability. Mol Biol Evol 30, 772–780.

Li, Z., Tiley, G.P., Galuska, S.R., Reardon, C.R., Kidder, T.I., Rundell, R.J., and Barker, M.S. (2018). Multiple large-scale gene and genome duplications during the evolution of hexapods. Proceedings of the National Academy of Sciences 201710791.

Löytynoja, A., and Goldman, N. (2008). Phylogeny-Aware Gap Placement Prevents Errors in Sequence Alignment and Evolutionary Analysis. Science 320, 1632–1635.

Lyons, E., Pedersen, B., Kane, J., and Freeling, M. (2008). The Value of Nonmodel Genomes and an Example Using SynMap Within CoGe to Dissect the Hexaploidy that Predates the Rosids. Tropical Plant Biology 1, 181–190.

Maere, S., De Bodt, S., Raes, J., Casneuf, T., Van Montagu, M., Kuiper, M., and Van de Peer, Y. (2005). Modeling gene and genome duplications in eukaryotes. Proceedings of the National Academy of Sciences 102, 5454–5459.

McKain, M.R., Tang, H., McNeal, J.R., Ayyampalayam, S., Davis, J.I., dePamphilis, C.W., Givnish, T.J., Pires, J.C., Stevenson, D.W., and Leebens-Mack, J.H. (2016). A Phylogenomic Assessment of Ancient Polyploidy and Genome Evolution across the Poales. Genome Biol Evol 8, 1150–1164.

Nielsen, R., and Yang, Z. (1998). Likelihood Models for Detecting Positively Selected Amino Acid Sites and Applications to the HIV-1 Envelope Gene. Genetics 148, 929–936.

Price, M.N., Dehal, P.S., and Arkin, A.P. (2010). FastTree 2 – Approximately Maximum-Likelihood Trees for Large Alignments. PLOS ONE 5, e9490.

Soderlund, C., Bomhoff, M., and Nelson, W.M. (2011). SyMAP v3.4: a turnkey synteny system with application to plant genomes. Nucleic Acids Res 39, e68.

Tiley, G.P., Barker, M.S., and Burleigh, J.G. Assessing the performance of Ks plots for detecting ancient whole genome duplications. Genome Biol Evol.

Van Bel, M., Diels, T., Vancaester, E., Kreft, L., Botzki, A., Van de Peer, Y., Coppens, F., and Vandepoele, K. (2018). PLAZA 4.0: an integrative resource for functional, evolutionary and comparative plant genomics. Nucleic Acids Res 46, D1190–D1196.

Vanneste, K., Van de Peer, Y., and Maere, S. (2013). Inference of Genome Duplications from Age Distributions Revisited. Mol Biol Evol 30, 177–190.

Vanneste, K., Sterck, L., Myburg, A.A., Peer, Y.V. de, and Mizrachi, E. (2015). Horsetails Are Ancient Polyploids: Evidence from Equisetum giganteum. The Plant Cell 27, 1567–1578.

Wang, Y., Tang, H., DeBarry, J.D., Tan, X., Li, J., Wang, X., Lee, T., Jin, H., Marler, B., Guo, H., et al. (2012). MCScanX: a toolkit for detection and evolutionary analysis of gene synteny and collinearity. Nucleic Acids Res 40, e49.

Yang, Z. (2007). PAML 4: Phylogenetic Analysis by Maximum Likelihood. Mol Biol Evol 24, 1586–1591.
